# A Text-Book upon the Pathogenic Bacteria for Students of Medicine and Physicians

**Published:** 1896-12

**Authors:** 


**A Text-book upon the Pathogenic Bacteria for Students of Medicine and Physicians.** By JOSEPH McFARLAND, M.D. Pp. 359. Philadelphia: W. B. Saunders. 1896.

This work has for its main object the description of the pathogenic bacteria in such a readable, but at the same time accurate, manner as to be of service to students, practitioners, and others whose daily work or inclination affords a motive for acquiring information upon this subject. It labours, therefore, under the disadvantage of losing the wealth of illustration afforded by a study of the non-pathogenic forms, and is likely to create difficulties for the practical worker by failing to prepare him for the recognition of the very numerous associates of the specific organisms which will confront him at the outset of any serious investigation. On the other hand, the limitation to practically "only such bacteria as can be proven pathogenic" dispenses with a large amount of matter which is not of direct interest to the class of readers for whom the work is specially intended; and this object would perhaps have been more perfectly realised if the second part only had been published, for here the material and the illustrations have been judiciously selected from the best sources.

The introduction to the first part consists of an historical account of the evolution of the science, commencing at what many will consider an unnecessarily early date. The apparently reasonable view that the consideration of bacteria should begin with their probable discoverer, Leeuwenhoek, is here stigmatised as folly; and the opportunity is taken to trace a somewhat forced connection with the very audacious speculations of the early Greek philosophers. The concise but clearly worded record of progress, with which this section closes, brings into great prominence the rapidity with which the specific organisms of the most important and widespread diseases were discovered when a start was once made.

The chapter on the composition and morphology of bacteria is not one of the happiest efforts. Nencki is credited with the discovery that "the chemical analysis of bacteria in the active state consists of 82.42 per cent. of water," and that mycoprotein "has the composition C 52.32, H 7.55, N 14.75." Whether this observer is in the habit of calculating his results on a basis of 74.62 parts, or cautiously leaves a margin of more than 25 per cent. for undiscovered elements, does not appear. But a still more striking instance of the danger of incorporating interesting pieces of information without due consideration occurs a page or two later. After being told that the weight of an average bacterium is $\frac{1}{10,000,000,000}$ milligramme (Nägeli), and that, doubling hourly, one germ will, under ideal circumstances, become in three days four thousand, seven hundred and seventy-two billions (Cohn), both of which statements are sufficiently near the truth, we learn that the "demnition total" would, according to a most eminent writer on the subject, "weigh no less than seventy-five hundred tons." At first sight it would seem that an explanation has at last been given of the exorbitant prices charged by scientific instrument makers for incubators. A broth culture measuring 10 c.c. often contains ten thousand million bacteria, weighing on this basis of calculation about thirty-five pounds. On a moderate computation the cotton wool plug will weigh as much, and the tube and culture medium a thousand (more probably twenty thousand) times as much as the bacteria. A pressure, therefore, of about fifteen tons will be brought to bear on the thin sheet metal of the incubator by each culture tube; and though it is true that in general the former is shielded by the bottom of a thin glass beaker supporting the tubes, it is not surprising that the effects of their enormous weight have to be counteracted by a subtle system of struts and stays at prodigious expense. On closer inspection, unfortunately, this explanation is found to be scarcely consistent with hard facts. If a bacterium weighs the one ten-thousand-millionth of a milligramme, the weight of ten thousand million will be one milligramme; of a billion, one decigramme; and the weight of four thousand, seven hundred and seventy-two billion bacteria will be 477.2 grammes, or slightly more than one pound. It is apparently impossible to deny the British origin of this poetic flight; but it seems a thousand pities that the story of the prolific germ did not find a more appropriate birth-place in the land of tall tales. A sentence on page 35 seems to imply that the development of a spore depends upon its being "accidentally" dropped into some nutrient medium; and page 40 contains a definition of animal and vegetable life considerably more simple than satisfactory.

The chapter on the physiological activities of bacteria is more satisfactory, though somewhat confused. A paragraph dealing with the reduction of nitrites (? nitrates) in particular completely misrepresents the result of Winogradsky's researches on the production of nitrates and nitrites.

The vexed question of immunity is on the whole well handled in so small a compass.

The methods of examination and culture of micro-organisms are clearly described, though in several details the practice recommended differs from that in force on this side the Atlantic, and is occasionally incorrect. Boiling gelatine media over a naked flame invariably impairs their setting powers; this is indeed proved by the direction to filter a litre of gelatine through a cold filter—quite an impossibility in this climate, with a nutrient gelatine which would subsequently stand an incubating temperature of 22° C. The practice of steeping the meat for some hours in water in the preparation of bouillon is almost universally discarded in favour of boiling up directly, by which means also the subsequent filtration is much expedited.

It is the second part of this work that will appeal most strongly to the readers to whom it is particularly addressed; and it is gratifying, therefore, to find a distinct improvement on the earlier chapters. The descriptions of the bacteria connected with the various specific diseases are concisely and clearly written; and a sufficiently detailed account is given of matters of interest, such as the preparation of anti-toxic serum, to enable students to intelligently follow modern developments. It is satisfactory to find unqualified approval of Löffler's medium for the cultivation of the bacillus diphtheriæ, and a note of the difficulty of growing this organism on agar or glycerin agar except by a process of education which renders these media totally unsuitable for diagnostic purposes. In connection with the first stage of the preparation of diphtheria antitoxin, it is stated that the author has successfully introduced the dose of toxin into the trachea, instead of, as usual, into the subcutaneous tissue of the animal. As the dose may amount to 300 c.c. this would seem a bold expedient. Under this section, as well as those dealing with tetanus and anthrax, credit is properly given to Brieger, Roux, Behring, and others, for their work on the toxins; but no mention is made of the very important researches of Sidney Martin on the products elaborated by these organisms.

The illustrations in this part are mostly excellent reproductions from the atlas of Fränkel and Pfeiffer. One obvious drawback to this system of collecting illustrations is that photographs are not invariably made to the same scale. Of this there is a striking example in the representations of the typhoid bacillus and bacillus coli, which are obviously intended to be compared, but which leave a very erroneous impression as to their relative size, owing to the different degrees of magnification.

